# Microdroplet-Mediated Radical Polymerization

**DOI:** 10.1021/acscentsci.2c00694

**Published:** 2022-08-12

**Authors:** Kyoungmun Lee, Hyun-Ro Lee, Young Hun Kim, Jaemin Park, Suchan Cho, Sheng Li, Myungeun Seo, Siyoung Q. Choi

**Affiliations:** ^†^Department of Chemical and Biomolecular Engineering, ^‡^Department of Chemistry, Korea Advanced Institute of Science and Technology (KAIST), Daejeon 34141, Republic of Korea; ∇ KAIST Institute for the Nanocentury, KAIST, Daejeon 34141, Republic of Korea

## Abstract

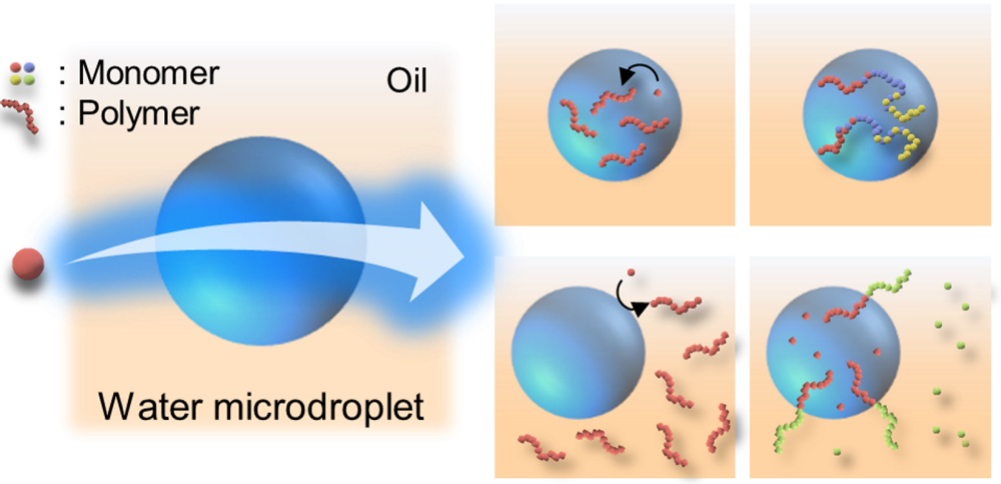

Micrometer-sized aqueous droplets serve as a unique reactor that drives various chemical reactions not seen in bulk solutions. However, their utilization has been limited to the synthesis of low molecular weight products at low reactant concentrations (nM to μM). Moreover, the nature of chemical reactions occurring outside the droplet remains unknown. This study demonstrated that oil-confined aqueous microdroplets continuously generated hydroxyl radicals near the interface and enabled the synthesis of polymers at high reactant concentrations (mM to M), thus successfully converting the interfacial energy into the synthesis of polymeric materials. The polymerized products maintained the properties of controlled radical polymerization, and a triblock copolymer with tapered interfaces was prepared by the sequential addition of different monomers into the aqueous microdroplets. Furthermore, a polymerization reaction in the continuous oil phase was effectively achieved by the transport of the hydroxyl radicals through the oil/water interface. This interfacial phenomenon is also successfully applied to the chain extension of a hydrophilic polymer with an oil-soluble monomer across the microdroplet interface. Our comprehensive study of radical polymerization using compartmentalization in microdroplets is expected to have important implications for the emerging field of microdroplet chemistry and polymerization in cellular biochemistry without any invasive chemical initiators.

## Abstract 2

Aqueous microdroplets spontaneously facilitate radical polymerizations, enabling the synthesis of hydrophilic polymer, triblock copolymer, hydrophobic polymer, and amphiphilic diblock copolymer.

## Introduction

Microdroplet chemistry is of particular interest as a powerful system to promote chemical reactions that are difficult to carry out in bulk phases without the use of catalysts.^1−10^ Compared to bulk-phase-mediated reactions, microdroplet-mediated reactions can enhance the reaction rate drastically by factors of ≥10^3^.^1−5^ The first quantitative demonstration of accelerated chemical reaction rate in water microdroplets was reported by Lee and co-workers.^5^ Moreover, it has been observed that several unique reactions, such as spontaneous reduction of organic molecules and metal ions,^6,7^ chemoselective N-alkylation of indoles,^8^ and generation of hydrogen peroxide (H_2_O_2_),^9,10^ can be effectively induced in micrometer-sized water droplets without catalysts while they scarcely appear in bulk aqueous solutions. These intriguing observations in the microdroplets are attributed to their interfacial properties, including the increase of the reaction rate constant in microcompartments^2^ and the high density^11^ and possible alignment of molecules near the droplet surfaces.^12^ Notably, a strong electric field (E⃗ ≈ 10^7^ V/cm) has been recently reported to be present at the surface of aqueous microdroplets^11^ which has the potential to produce hydroxyl radicals from water molecules.^9^

Despite the ability of microdroplets to facilitate unusual chemical reactions, these reactions have been limited to the synthesis of low molecular weight compounds (molecular weight <1000 g/mol)^1−10^ at trace amounts of reactants (nM to μM). No study has yet demonstrated the utilization of microdroplets to generate high molecular weight polymer products at high reactant concentrations (mM to M). An inherent limitation of the commonly used sprayed aqueous microdroplet system is its short lifetime. Evaporation of the microdroplets rapidly occurs at the air/water interfaces, preventing the retention of micrometer-sized droplets for long periods. Furthermore, the absence of the microdroplet reservoir also makes it challenging to investigate any chemical reactions that may occur outside the droplet. We hypothesize that if aqueous microdroplets can be prepared to continuously exist in an oil phase, then such systems may overcome the restrictions associated with the sprayed aqueous microdroplets and be used to conduct reactions requiring microdroplets with an extended lifetime.

**Figure 1:**
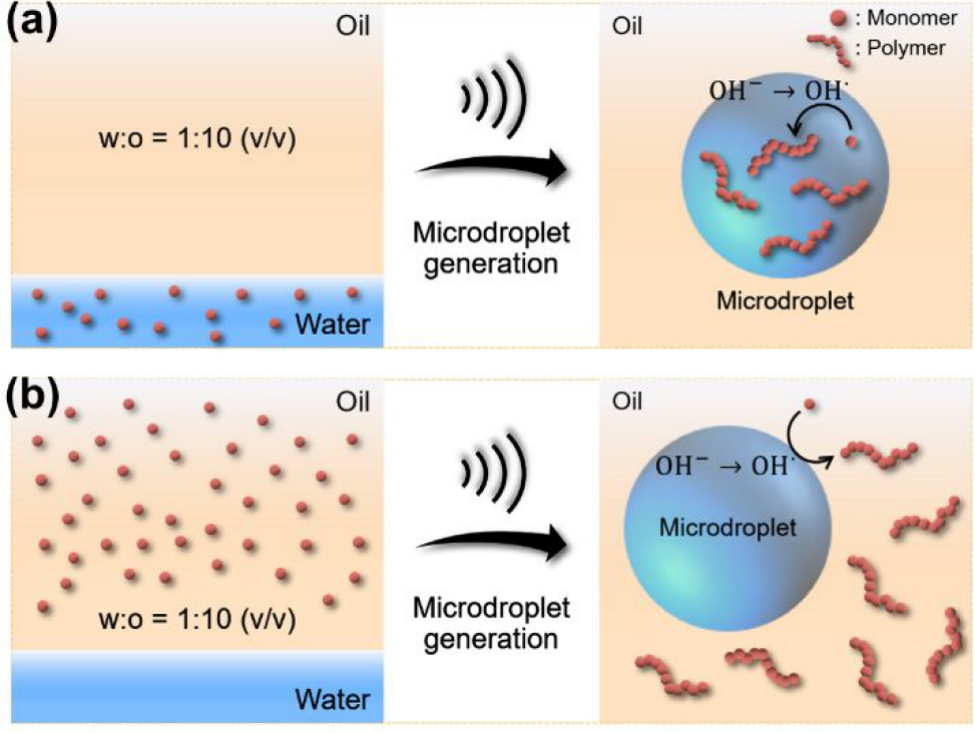
Microdroplet-mediated radical polymerization. We emulsified 1:10 (v/v) mixtures of water and hexadecane solutions via ultrasound irradiation. A strong electric field formed near the oil/water interface and then induced the continued formation of hydroxyl radicals which could initiate radical polymerization in both (a) the dispersed water and (b) the continuous oil phases without additive chemical initiators.

Here, we demonstrated that by utilizing an oil/water interface instead of an air/water interface to construct microdroplets, the reaction time scale can be extended from microseconds to hours. The produced microdroplets continuously generated hydroxyl radicals near the oil/water interface, enabling the successful synthesis of polymers via successive chemical reactions (Figure 1). When the polymerization reactions occurred in the presence of a reversible addition–fragmentation chain transfer (RAFT) agent, the synthesized polymers exhibited the characteristics of controlled radical polymerization for various types of monomers. A triblock copolymer with tapered interfaces was also produced by sequentially adding different monomers to aqueous microdroplets. The polymerization in the continuous oil phase can be induced by the transport of the hydroxyl radicals into the oil phase. Chain extension of a hydrophilic polymer with an oil-soluble monomer across the interface was also achieved. In contrast, no polymerization reaction occurs in bulk solutions. The demonstration of oil-confined aqueous microdroplet reactors may provide green pathways for synthesizing high molecular weight products in cells by biomolecular reactions confined to micrometer-sized reactors without enzymes or catalysts. Additionally, the combination of the strengths of our system and the uniqueness of the aqueous microdroplets has significant implications for the emerging field of microdroplet chemistry.

## Results and Discussion

### Generation of H_2_O_2_ in Oil-Confined Aqueous Microdroplets

**Figure 2:**
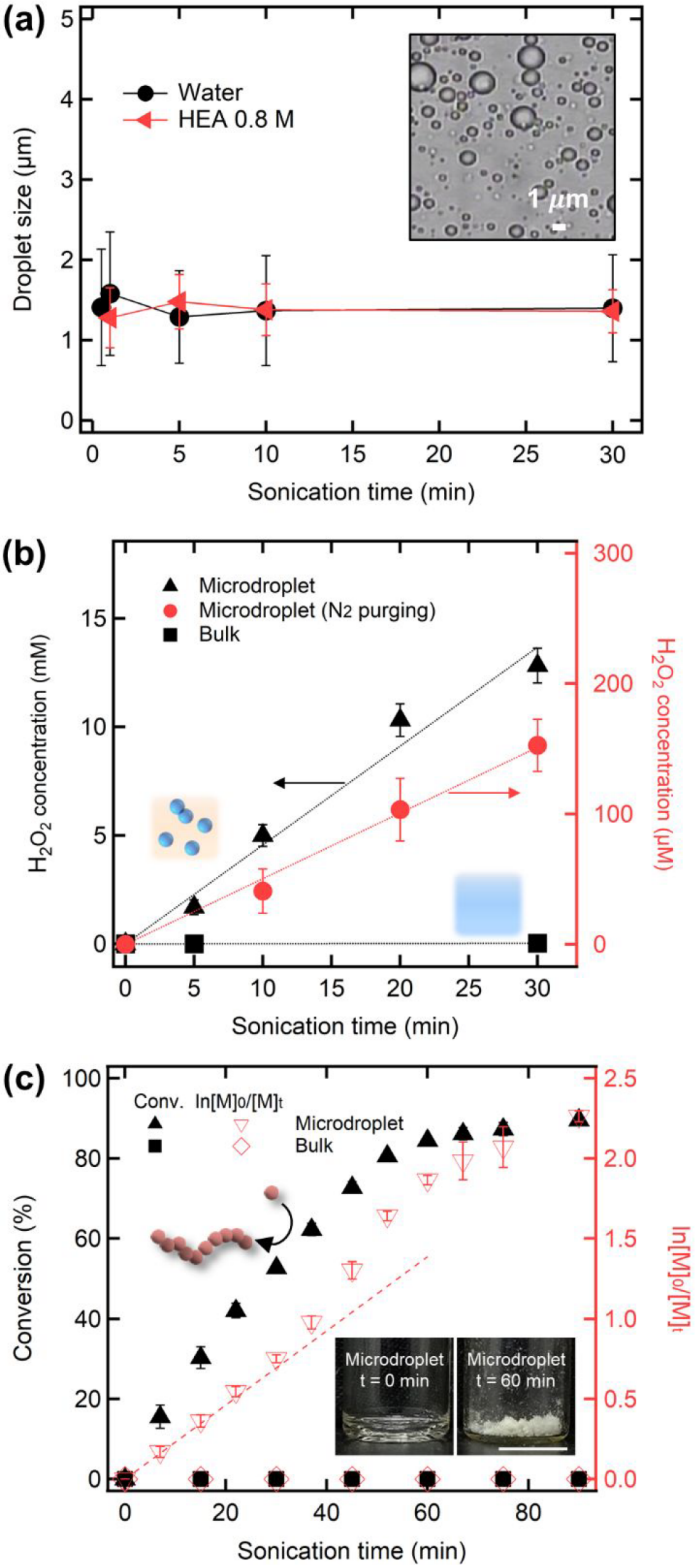
(a) Diameter of the microdroplets generated by ultrasonic emulsification of 1:10 (v/v) mixtures of water solutions and hexadecane oil as a function of sonication time. The inset image shows generated aqueous microdroplets at 30 min without HEA. (b) Concentration of H_2_O_2_ produced in the microdroplets increased with the ultrasound irradiation. The H_2_O_2_ concentration was below the detection limit in a bulk water environment. (c) Polymerization of HEA within the aqueous microdroplets closed by hexadecane oil ([HEA] = 0.8 M, [HEA]:[TTC] = 300:1). Conversion linearly increases under continuous ultrasonic irradiation, not following first-order kinetics (dotted line, generated by the first two points). The bulk HEA solution data is also presented as a reference. The inset images are the remaining substance after removal of solvents (scale bar: 1 mm).

We first investigated the spontaneous generation of H_2_O_2_ in aqueous microdroplets enclosed by oil. It was motivated by Xiong and co-workers,^11^ who reported the observation of a strong electric field (E⃗ ≈ 10^7^ V/cm) at the oil-confined microdroplet surface. This electric field strength is sufficient to produce hydroxyl radicals from hydroxide ions,^9,10^ which readily recombine to form H_2_O_2_.^13^ We emulsified 1:10 (v/v) mixtures of water and hexadecane through an ultrasonic bath to generate micrometer-sized aqueous droplets. The created aqueous microdroplets of water-in-oil emulsion exhibited a size of ca. 1.5 μm in diameter (Figures 2a and S1a).

We utilized a spectroscopic method^14^ to quantify the amount of generated H_2_O_2_ in the aqueous droplets collected by centrifugation (Figure S2). As shown in Figure 2b, the concentration of H_2_O_2_ increased linearly with the ultrasound irradiation time and exceeded 10 mM within 30 min. This value is almost 100 times higher than that reported in previous microdroplet studies generating H_2_O_2_ by the air/water interface (<35 μM).^9,10^ The deviation may be attributed to the ability of our system to retain the microdroplet structure for hours. The continued creation of newly formed bare water interfaces via ultrasound can further contribute to the increase in the H_2_O_2_ concentration. In contrast, H_2_O_2_ was not detected when bulk water was sonicated, indicating that the microdroplet system, not ultrasound energy, mainly contributes to the production of H_2_O_2_.

We further evaluated H_2_O_2_ concentration after removal of the dissolved O_2_ by N_2_ purging. A relatively high concentration of H_2_O_2_ (>150 μM at 30 min) was still observed when N_2_-purged mixtures of water and hexadecane were emulsified (Figure 2b) though the concentration of H_2_O_2_ was decreased. This suggests that O_2_ may significantly enhance H_2_O_2_ production as previously observed.^10^ All of the observations above indicate that the interfacial energy of the microdroplets trapped in the oil phase could be exploited to generate H_2_O_2_, which might be produced by the recombination of hydroxyl radicals capable of initiating radical polymerization.

### Microdroplet-Mediated Aqueous RAFT Polymerization

To estimate the viability of the microdroplet-mediated polymerization reaction in aqueous media, we first conducted a model RAFT polymerization with 2-hydroxyethyl acrylate (HEA) as the water-soluble monomer and S,S′-bis(α,α′-dimethyl-α″-acetic acid)trithiocarbonate (TTC) as the RAFT agent. An aqueous solution of HEA (0.8 M) and TTC ([TTC] = [HEA]/300) was prepared, and the 1:10 (v/v) mixture of the aqueous solution and hexadecane was irradiated with ultrasound. The diameter of the generated droplets was ca. 1.5 μm (Figures 2a and S1b), similar to that of the pure water microdroplets.

Encouragingly, over time we observed the formation of polymers in the oil-confined aqueous microdroplets, while the monomers remained unpolymerized in the bulk water (Figure 2c). Conversion of the monomer linearly increased and reached greater than 80% within an hour. The polymerization rate in the microdroplets showed an order similar to that of radical polymerization in the bulk; however, it did not follow the first-order kinetics for the monomer concentration typically observed in controlled radical polymerizations. This might be partially attributed to the reduced tension at the oil/water interface in the presence of HEA (Figure S3). As HEA is mostly consumed near the interface, where polymerization is initiated, the monomer concentration near the interface is expected to be higher than that in the bulk up to a critical conversion. The non-first-order kinteics may also be coupled to the continuous radical generation from the interface during ultrasonication. The molecular weight of the synthesized polymer (*M*_n,SEC_) determined by size exclusion chromatography (SEC) also gradually increased with the sonication time and was consistent with the theoretical molecular weight (*M*_n,th_) as shown in Figure S4. We further demonstrated the consistency between *M*_n,th_ and the molecular weight calculated by comparing the proton signals of the RAFT agent and the polymer backbone in the ^1^H NMR spectra (*M*_n,NMR_) in Table S1. Side reactions, including chain cleavage, were not observed by ^1^H NMR spectroscopy (Figure S5a) and SEC (Figure S5b). The polymerization rate decreased in the presence of 4-methoxyphenol as a radical scavenger^15^ (Figure S6), confirming that radical species initiate the RAFT polymerization within the microdroplets.

The concentration of active growing radicals during microdroplet polymerization was estimated by conducting free radical polymerization (FRP) of the HEA monomer. Under the steady-state assumption that the initiation and termination rates are identical, the propagation rate *R*_p_ = −d[M]/d*t* = *k*_p_[M][R·]_st–st_, where *k*_p_ is the propagation rate constant and [R·]_st–st_ is the concentration of the steady-state active radical chains. Using the known *k*_p_ value of HEA^16^ and the other measurable variables of *R*_p_ and [M] (Figure S7a), [R·]_st–st_ was estimated to be about 10^–8^ mol/L (Figure S7b), which is consistent with the typical radical concentration found in chain radical polymerization.^17^

We further tested the microdroplet-mediated polymerization by changing the monomer concentration and targeted degrees of polymerization, and we summarized the results in Table 1 (see Figure S8 for the SEC traces). HEA was successfully polymerized over a range of monomer concentrations, and control of the molecular weight was possible by varying the [HEA]:[TTC] ratio. The dispersity values were higher than that typically seen in controlled radical polymerization and decreased with the increasing TTC concentration. A relatively small dispersity of 1.29 was obtained at the highest TTC concentration tested (entry 5). Given that the radical concentration generated at the interface may not vary much, the high RAFT agent concentration seems to facilitate the degenerative chain transfer process between the growing chains.

**Table 1:** Microdroplet-Mediated Polymerization of Water-Soluble Monomers

entry	monomer^a^	[monomer] (M)	[M]_0_/[TTC]_0_	*t* (min)	conv. (%)^b^	*M*_n,SEC_ (Da)^c^	*M*_n,th_ (Da)^d^	*M*_w_/*M*_n_^c^
1	HEA	0.2	300	60	63	29 900	22 200	1.64
2	HEA	0.4	300	60	78	31 300	27 500	1.49
3	HEA	0.8	300	60	84	26 500	29 500	1.38
4	HEA	1.6	300	60	74	21 800	26 100	1.29
5	HEA	0.8	500	60	73	51 700	42 700	1.44
6	HEA	0.8	700	60	79	63 100	64 500	1.55
7	HEA	0.8	1000	60	80	92 700	93 200	1.76
8	AM	0.8	300	60	80	6 000	34 200	1.51
9	DMA	0.8	300	60	77	1 800	23 200	1.51
10	OEGMEMA	0.2	300	60	60	108 600	90 300	1.58

a HEA, 2-hydroxy ethyl acrylate; AM, 4-acryloylmorpholine; DMA, N,N-dimethylacrylamide; OEGMEMA, oligo(ethylene glycol) methyl ether methacrylate (*M*_n_ 500).

b Conversion of monomers was determined via ^1^H NMR spectroscopy.

c Analyzed based on poly(methyl methacrylate) standards with a flow rate 0.6 mL/min of 0.05 M LiBr dissolved dimethylformamide as an eluent at 50 °C.

d *M*_n,th_ is defined as *M*_n,th_ = conversion × [M]_0_/[TTC]_0_ × MW_monomer_ + MW_TTC_.

Other water-soluble monomers, including N,N-dimethylacrylamide (DMA), 4-acryloylmorphine (AM), and oligo(ethylene glycol) methyl ether acrylate (OEGMEMA), were also polymerized, supporting the broad applicability of the microdroplet-mediated radical polymerization. The discrepancy between the theoretical values and *M*_n,SEC_ calibrated based on the poly(methyl methacrylate) standards may come from the variation in hydrodynamic sizes of the polymers in the eluent.

To analyze the structures of the synthesized polymers, we conducted an end-group analysis of poly(N,N-dimethylacrylamide) (PDMA) oligomers synthesized by microdroplet-mediated RAFT polymerization through matrix-assisted laser desorption/ionization mass spectrometry (Figure S9). The PDMA oligomers were chosen for their excellent solubility in the matrix-soluble solvent. The oligomers were obtained by turning off the polymerization after 5 min of sonication. A regular interval of 99.10 in the mass-to-charge ratio (*m*/*z*) corresponds to a single DMA monomer unit (99.13 g/mol). The major series of peaks were assigned to PDMA containing the RAFT agent fragments at the chain ends. For instance, in Figure S9a, the peak at *m*/*z* = 976.77 agrees well with the calculated mass of PDMA with the degree of polymerization of 7. In Figure S9b, hydroxyl radical-derived chains also appeared at lower [TTC] ([TTC] = [DMA]/1000), which were generally observed at a low [chain transfer agent]-to-[initiator] ratio.^18−20^

### On/Off Control of the Microdroplet-Mediated Polymerization

**Figure 3:**
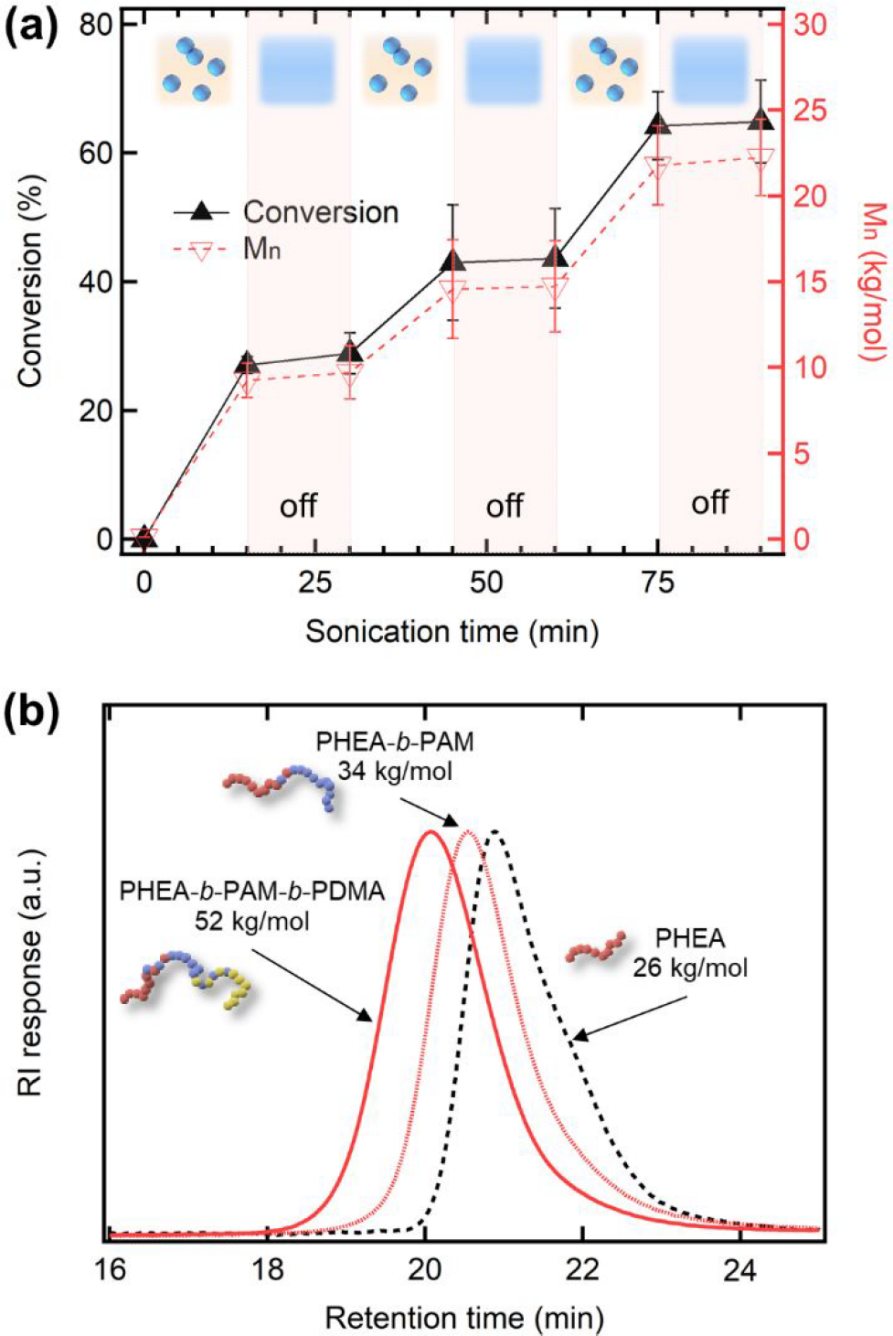
(a) Monomer conversion and molecular weight with altering ultrasonication (“on”) and centrifugal merging (“off”). The 1:10 (v/v) mixture of the HEA aqueous solution (300 equiv per TTC, [HEA] = 0.8 M) and hexadecane oil was used. (b) Dimethylformamide SEC traces before and after the addition of second (AM) and third monomers (DMA). PHEA-*b*-PAM-*b*-PDMA tapered triblock copolymer was synthesized via the addition of second and third monomers after 1 h of sonication at each stage (300 equiv per TTC, [HEA]_0_ = 0.8 M).

We further evaluated the temporal control over polymerization by utilizing the formation and coalescence of microdroplets. We found that the polymerization was stopped when ultrasound was switched off and the microdroplets were merged by centrifuging. Irradiation of the reaction mixture with ultrasound restarted polymerization. This on/off procedure was repeated three times at a 15 min interval to result in a step-like increase in conversion and molecular weight (Figure 3a). The reversible activation and deactivation of polymerization resemble the on–off behavior reported for photo- and sono-RAFT polymerizations.^21−24^ For the sono-RAFT polymerizations, the short lifetime of active hydroxyl radicals has been attributed to deactivation in the absence of ultrasound.

Utilizing the reversible activation of the microdroplet-mediated polymerization system, we synthesized a triblock copolymer by the sequential addition of different monomers without purification steps. After irradiating the reaction mixture containing HEA with ultrasound for 1 h, we added AM as a second monomer and sonicated for 1 h. The addition of DMA and sonicating for another 1 h successfully produced the triblock copolymer (Figures 3b and S10a). While the block interface would be tapered because the monomer was not fully consumed before the subsequent monomer addition, the SEC traces showed a clear shift to a higher molecular weight per addition.

### Microdroplet-Mediated Radical Polymerization of Oil-Soluble Monomers

**Figure 4:**
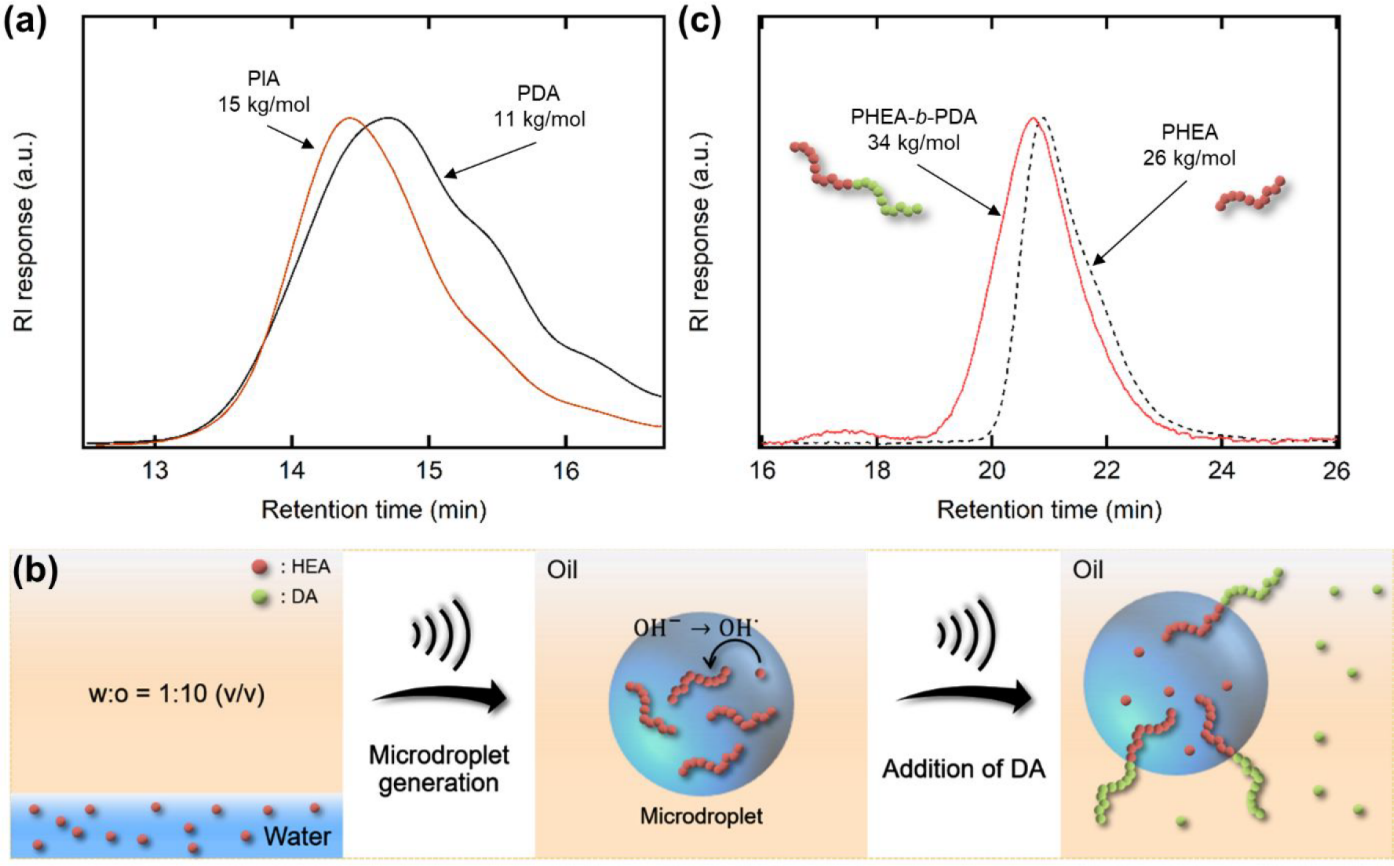
(a) Representative tetrahydrofuran SEC traces of the synthesized PDA and PIA ([monomer]_0_ = 0.4 M, 120 min sonication time). (b) Synthesis of PHEA-*b*-PDA. After polymerization of HEA (300 equiv per TTC, [HEA]_0_ = 0.8 M) by sonication for 1 h, chain extension was achieved by DA (0.8 M) addition followed by sonication for 2 h. (c) Representative dimethylformamide SEC traces of PHEA and PHEA-*b*-PDA.

We evaluated the possibility of FRP in the continuous oil phase initiated by the transport of hydroxyl radicals through the oil/water interface. Dodecyl acrylate (DA) and iso-decyl acrylate (IA) were tested as oil-soluble monomers. The 1:10 (v/v) mixtures of water with hexadecane solutions of DA or IA (0.4 M) were prepared and subjected to ultrasound for 2 h. Both monomers were successfully polymerized (Figure 4a). Since both DA and IA are insoluble in water, the generated hydroxyl radicals are assumed to meet the oil-soluble monomers at the interface and initiate polymerization.

We further investigated the chain extension of a hydrophilic polymer synthesized in the aqueous microdroplets with an oil-soluble monomer in the continuous phase (Figure 4b). We first ultrasonicated a mixture of hexadecane with an aqueous solution of HEA (0.8 M) and TTC ([TTC] = [HEA]/300) for 1 h. Then DA (0.8 M) was added to hexadecane, and an additional 2 h sonication was applied. Although the two monomers were strongly partitioned in the immiscible water and oil phases, chain extension across the interface produced a PHEA-*b*-PDA amphiphilic diblock copolymer as evidenced by the appearance of PDA protons in the ^1^H NMR spectrum (Figure S10b) and also a shift to higher molecular weight in the SEC trace (Figure 4c). We note that a small intensity at 17.5 min may correspond to PHEA-*b*-PDA aggregates because of poor solubility of the PDA block in dimethylformamide eluent.

### Comparative Study of Microdroplet-Mediated Radical Polymerization

Although we utilized an ultrasonic bath to generate microdroplets, the mechanism of the microdroplet-mediated polymerization is conceptually different from other sono-radical polymerizations.^21,25−28^ Previous studies have either converted sound energy to mechanical energy^25,26^ or utilized high-frequency ultrasound^21,27,28^ to initiate radical polymerization. For example, Wang et al.^26^ applied mechanical stress to piezoelectric particles using sound energy to generate radical species and initiate polymerizations. McKenzie et al.^21^ utilized high-frequency (∼400 kHz) ultrasound to produce hydroxyl radicals from water molecules by strong chemical effects. However, in our microdroplet-mediated polymerization, interfacial energy between two immiscible liquids was exploited to generate radicals. A strong electric field (E⃗ ≈ 10^7^ V/cm) at the oil-confined microdroplet surface^11^ produced hydroxyl radicals from hydroxide ions^9,10^ under mild conditions. As we observed, in our reaction system, ultrasound was only used to create oil-confined microdroplets without chain initiation or chain scission. This moderate system may be connected to important implications of cellular biochemistry to synthesize polymers.

Moreover, the initiation mechanism of the microdroplet-mediated radical polymerization could suggest a generic polymerization scheme that does not need consideration of solubility issues. Continuously generated hydroxyl radicals at the oil/water interface spontaneously initiate radical polymerization in both the dispersed water and the continuous oil phases. This interfacial phenomenon also could be used to synthesize amphiphilic diblock copolymers without the need for a cosolvent and additional purification processes, which are critical factors for the synthesis of amphiphilic diblock copolymers.^29^

## Conclusions

In summary, we demonstrated that microdroplet chemistry can be advanced to the synthesis of polymeric materials. The development of micrometer-sized aqueous reactors enclosed by oil can extend reaction time scales from microseconds to hours and continuously produce hydroxyl radicals, allowing for successful polymerization reactions. Through the microdroplet-mediated RAFT polymerization, the evolution of polymer chain length increases linearly with monomer conversions and reaction time, both of which are hallmarks of general controlled radical polymerization. The polymerization kinetic is non-first-order, possibly due to the higher monomer concentration near the microdroplet interface where polymerization is initiated. Given the long lifetime of microdroplets, we were also able to synthesize a PHEA-*b*-PAM-*b*-PDMA triblock copolymer with tapered interfaces by successive addition of each monomer. Furthermore, the transferred hydroxyl radicals through the oil/water interface facilitate the polymerization in the continuous oil phase. A PHEA-*b*-PDA amphiphilic diblock copolymer across the microdroplet interface was also effectively synthesized without the use of a cosolvent and subsequent purification steps. In more general terms, our findings on oil-confined microdroplet-mediated radical polymerization may have relevant implications for the emerging field of microdroplet chemistry. Chemical reactions unique in aqueous microdroplets may be broadened to include both the inner and outer droplet interfaces. Moreover, the increased microdroplet lifetime of this technique can be exploited to overcome low yield issues inherent in sprayed aqueous microdroplets. This study might also be related to cellular biochemistry for the synthesis of high molecular weight products, such as polymers, without any detrimental chemical initiators. The noncatalytic reactions in microdroplets may provide insight into how high molecular weight building blocks are created in the prebiotic era.

## Supporting Information Available

Additional details on materials; methods; and experimental results, including ^1^H NMR spectra, SEC traces, and matrix-assisted laser desorption/ionization mass spectrometer results (PDF)
Transparent Peer Review report available (PDF)
