# Supplementary material for: Microdroplet-Mediated Radical Polymerization
Source: ACS Cent Sci. 2022 Aug 12;8(9):1265–71. doi: 10.1021/acscentsci.2c00694 (PMC9523774; doi:10.1021/acscentsci.2c00694)
Supplement: Supplementary file 1 — oc2c00694_si_001.pdf [file oc2c00694_si_001.pdf]

Supporting Information

Microdroplet-Mediated Radical Polymerization

Kyoungmun Lee,[a] Hyun-Ro Lee,[a] Young Hun Kim,[a] Jaemin Park,[a] Suchan Cho,[b] Sheng Li,*[,a,c] Myungeun Seo,*[,b,c] and Siyoung Q. Choi*[,a,c]

[a]Department of Chemical and Biomolecular Engineering, Korea Advanced Institute of Science and Technology (KAIST), Daejeon 34141, Korea

[b]Department of Chemistry, KAIST, Daejeon 34141, Korea

[c]KAIST Institute for the Nanocentury, KAIST, Daejeon 34141, Korea

*E-mail: shengli@kaist.ac.kr (Li, S.); seomyungeun@kaist.ac.kr (Seo, M.); sqchoi@kaist.ac.kr (Choi, S. Q.)

This PDF file includes:

Materials and Method

Supporting Figures S1-10

Supporting Table S1

Supporting References

Materials and Methods

Materials

2-Hydroxyethyl acrylate (HEA, 96%, Sigma-Aldrich) was washed with hexane 5 times to remove divinyl impurities,[1] then passed through alumina column to remove inhibitors before use. The RAFT agent S,S'-bis(α,α'-dimethyl-α''-acetic acid)trithiocarbonate was synthesized via previously reported strategy[2] and analyzed by ^{1}H and ^{13}C NMR spectroscopy. N,N-dimethylacrylamide (DMA, 99%), 4-acryloylmorpholine (AM, 97%), and oligo(ethylene glycol) methyl ether methacrylate (OEGMEMA, M_n 500 g/mol) were purchased from Sigma-Aldrich. Dodecyl acrylate (DA, 98%) and isodecyl acrylate (IA) were purchased from Tokyo Chemical Industry. All the monomers passed through alumina column before use to remove contained inhibitors. Hydrogen peroxide (H_2O_2) solution (35%) was purchased from Junsei Chemical. Hexadecane (99%), 4-methoxyphenol (MEHQ), sodium hydroxide (≥ 97%), potassium iodine (≥ 99%), ammonium molybdite tetrahydrate (≥ 99.98%), and potassium hydrogen phthalate (≥ 99.5%), were all purchased from Sigma-Aldrich and used without further purification. Ultrapure water was produced by Millipore ICW-3000 water purification system (> 18 MΩ).

Microdroplet generation

Aqueous microdroplets were generated by the ultrasonic water bath (WUC-D06H, DAIHAN scientific) which maintains an almost constant water level and temperature (25-28 ℃) via continuous circulations. The reaction vial was placed at a depth of about 7.5 cm from the transducer plate. The reaction samples (200 μl water solutions and 2 ml hexadecane solutions in 15 ml vial (Samwoo Science)) were subjected to ultrasonication (frequency of 40 kHz and 100% of maximum amplitude) for the desired reaction time to start radical polymerization or to generate H_2O_2. The size of produced microdroplets was observed by optical microscopy with CCD camera (WAT-902H, Watec) and analyzed by ImageJ.

Quantification of generated H_2O_2

The concentration of H_2O_2 in aqueous microdroplets according to sonication time was determined via spectroscopic method.[3] To investigate the effect of solvent dissolved O_2, N_2 purged and non-purged samples were prepared. For N_2 purged sample, both water and oil were degassed with nitrogen for 15 min before microdroplet generation. After desired irradiation time of ultrasound, aqueous solutions were collected by centrifugation (14,500 rpm for 5 min, MiniSpin Plus, Eppendorf) and we quantified H_2O_2 concentration. The I^- ion, which has no absorption peak, is oxidized to I_3^- ion having

absorption peak at 353 nm[4] from the catalytic activity of ammonium molybdite under the presence of H_2O_2. In detail, 100 µl of two solutions, A (0.4 M KI, 0.1 M NaOH, and 0.02 mM ($(NH_4)_6Mo_7O_{24}$) and B (0.1 M $C_8H_5KO_4$), were mixed with 100 µl of a diluted sonicated sample and analyzed through the absorption peak at 353 nm using UV-vis spectrometer (UV-2600, Shimadzu).

Microdroplet-mediated radical polymerizations

For the synthesis of homopolymers, desired monomer concentrations were prepared either in water or hexadecane oil. Before the reaction mixtures were irradiated, all samples were degassed with nitrogen for 15 min. The reaction samples (200 µl water solutions and 2 ml hexadecane solutions in 15 ml vial) were subjected to ultrasonication (frequency of 40 kHz and 100% of maximum amplitude) for the desired reaction time.

PHEA-*b*-PAM-*b*-PDMA tapered triblock copolymer was synthesized through the following steps. After applying ultrasound to reaction samples of 2 ml hexadecane and 200 µl aqueous solutions of HEA (0.8 M) and TTC ([TTC] = [HEA]/300) for 1 h, we added AM (20 µl) and irradiated ultrasound for 1 h. Finally, DMA (15 µl) was added as a third monomer and sonicated for an additional 1h.

To synthesize PHEA-*b*-PDA amphiphilic diblock copolymers, we first prepared aqueous solutions of HEA (0.8 M) and TTC ([TTC] = [HEA]/300) and irradiated ultrasound to the 1:10 (v/v) mixtures of aqueous solutions and hexadecane. After 1 h reaction, we added 400 µl DA to the solutions and an additional 2 h sonication was applied.

On/off experiments

The reaction mixtures of 2 ml hexadecane and 200 µl aqueous solutions of HEA (0.8 M) and TTC ([TTC] = [HEA]/300) submerged in the ultrasonic bath and subjected to ultrasonication to turn on the polymerization reaction. After 15 min, ultrasonication was switched off and the emulsified reaction mixtures were centrifuged (5,000 rpm for 5 min, Supra 22k, Hanil) to turn off microdroplet-mediated RAFT polymerizations by merging droplets. This on/off processes were repeated 3 times at 15-minute intervals.

Interfacial tension measurement

The interfacial tensions of water/hexadecane in the presence of HEA monomer were investigated by a pendant drop technique.[5] A 10 µl HEA aqueous solution droplets was produced at the end of a micro-syringe tip (Outer diameter = 0.72 mm, Hamiltonian) that was immersed in the hexadecane oil. Droplet images were recorded at time interval of 30 seconds using a CCD camera (WAT-902H, Watec), and the interfacial tension were obtained by analyzing the shapes of the droplets.

Characterizations of microdroplet synthesized polymers

The number average molecular weight (M_n), weight average molecular weight (M_w), and dispersity (M_w/M_n) of homopolymers and block copolymers were analyzed by size exclusion chromatography (SEC, YL 9100 HPLC system, YL instrument) with a flow rate 0.6 ml/min of 0.05 M LiBr dissolved dimethylformamide (DMF) as an eluent at 50 ℃. The SEC instrument consists of 2 × Shodex GF-7M HQ 9 μm columns (7.5 × 300 mm), Shodex GF-1G 7B Guard 9 μm (7.5 × 50 mm), and refractive index detector (YL 9170 RI detector, YL instrument). The system was calibrated using Shodex Standard SL-105 (molecular weight ranges from 500 g/mol to 22,000 g/mol) and SM-105(molecular weight ranges from 1,200 g/mol to 2,700,000 g/mol). The M_n, M_w and M_w/M_n of oil-soluble polymers were determined by SEC (Agilent 1260 Infinity II) analysis with a flow of 1 mL/min of tetrahydrofuran (THF) as eluent at 35 ℃. The SEC instrument consists of 2 × Agilent PLgel 5 μm MIXED-C columns (300 × 7.5 mm), 1260 Infinity II Quaternary pump, and 1260 Infinity II MDS refractive index detector. The system was calibrated using Agilent PS-M EasiVial calibration kit (molecular weight range: 162–364,000 g/mol). Conversion of monomer was calculated from ^{1}H NMR spectra using 400 MHz Bruker Avance III HD (9.4 T) operating at ambient temperature in desired solvent.

The matrix-assisted laser desorption/ionization time-of-flight mass spectrometry (MALDI-TOF) measurements were performed on a Bruker Autoflex maX instrument equipped with a 355 nm nitrogen laser with a 20 kV acceleration voltage. The positive ions were detected in the reflector mode. MALDI-TOF samples were prepared by mixing poly(dimethylacrylamide) (PDMA) oligomer (1.5 mg/mL, 10 μL) and the matrix (trans-3-indoleacrylic acid, 10 mg/mL, 90 μL) in THF without salts.

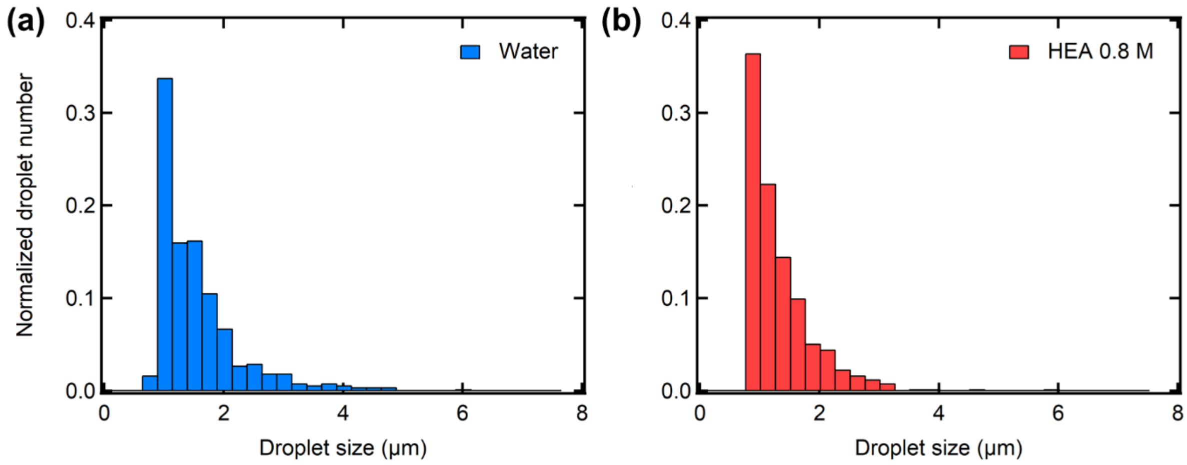

Figure S1. Size distribution of the microdroplets generated by ultrasonic emulsification of 10:1 (v/v) mixtures of hexadecane oil and (a) pure water or (b) HEA solution at 30 min sonication time.

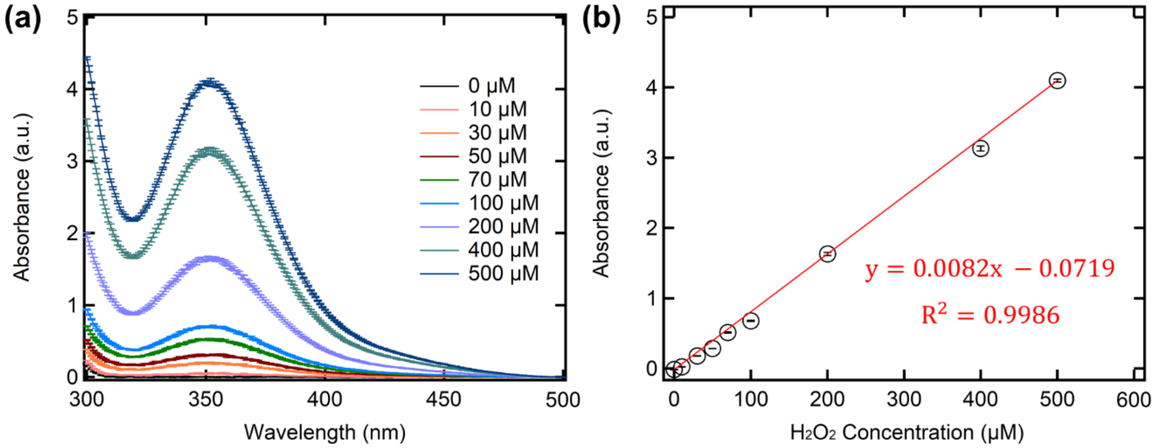

Figure S2. Intensity of the UV-visible absorption according to the H_2O_2 concentration in a bulk water. The I^- ion is oxidized to I_3^- ion from the catalytic activity of ammonium molybdite under the presence of H_2O_2, and absorption peak occurs at 353 nm. (a) UV-vis spectra of the solutions with varying concentration of H_2O_2. (b) Linear increase of absorbance peak at 353 nm with increase of H_2O_2 concentration. Higher concentrations above 500 **μ**M were measured after dilution.

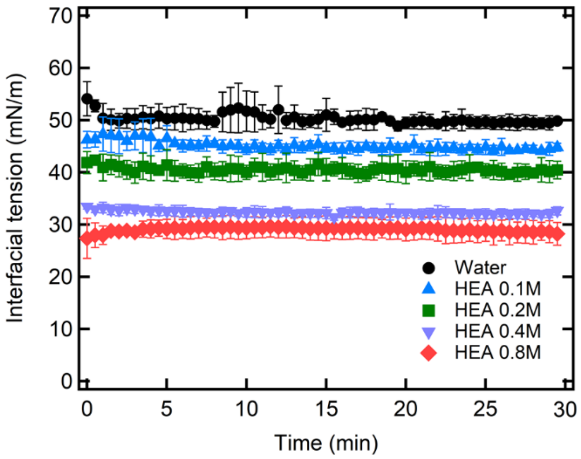

Figure S3. Interfacial tension of water/hexadecane with desired concentration of HEA. The interfacial tensions are reduced in the presence of HEA.

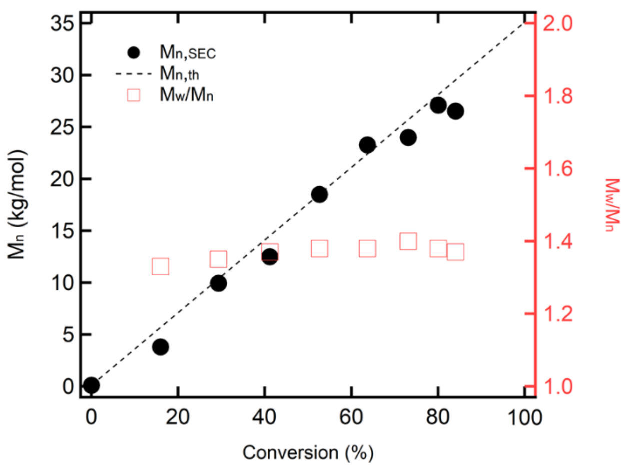

Figure S4. Characterization of the PEHA synthesized via microdroplet-mediate RAFT polymerization. ($[HEA]_0$ = 0.8 M, $[HEA]_0/[TTC]_0$ = 300). Molecular weight of PHEA increases linearly with the conversion of HEA monomer. $M_{n,SEC}$ follows $M_{n,th}$ ($M_{n,th}$ = (conversion × $[M]_0/[TTC]_0$ × $MW_{monomer}$ + MW_{TTC}).

Table S1. Characterization of PHEA synthesized by microdroplet-mediated RAFT polymerization.

Monomer[a]	t (min)	Conv. (%)[b]	$M_{n,SEC}$ (Da)[c]	$M_{n,th}$ (Da)[d]	$M_{n,NMR}$ (Da)[e]
HEA	7	16	3,800	5,800	4,700
HEA	15	29	9,900	10,500	10,900
HEA	22	41	12,500	14,600	12,500
HEA	30	53	18,500	18,600	17,500
HEA	37	64	23,300	22,500	23,500
HEA	45	73	24,000	25,800	25,600
HEA	52	80	27,100	28,200	27,400
HEA	60	84	26,500	29,500	28,100
HEA	67	85	27,500	29,900	28,600
HEA	75	89	27,800	31,100	29,800
HEA	90	90	27,900	31,600	30,100

[a]$[HEA]_0$ = 0.8 M, $[HEA]_0/[TTC]_0$ = 300. [b]Conversion of monomers was determined via ^{1}H NMR spectroscopy. [c]Analyzed based on poly(methyl methacrylate) standards with a flow rate 0.6 ml/min of 0.05 M LiBr dissolved DMF as an eluent at 50 °C. [d]$M_{n,th}$ is defined as $M_{n,th} = \text{conversion} \times [M]_0/[TTC]_0 \times MW_{monomer} + MW_{TTC}$. [e]Calculated by comparing the proton signals of the RAFT agent and the polymer backbone in ^{1}H NMR spectra.

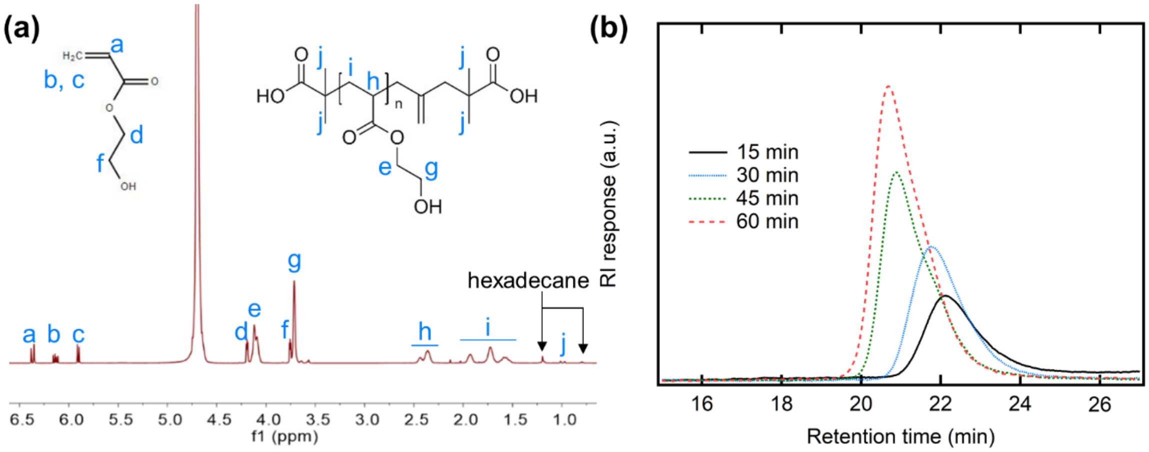

Figure S5. (a) ^{1}H NMR spectra of PHEA (300 equivalent per TTC, $[HEA]_0$ = 0.8 M, 60 min sonication time) synthesized via microdroplet-mediated RAFT polymerization (400 MHz, D_2O, 25 °C). (b) Representative SEC traces depicting the evolution of molecular weight with reaction time during the microdroplet polymerization of HEA (300 equivalent per TTC, $[HEA]_0$ = 0.8 M).

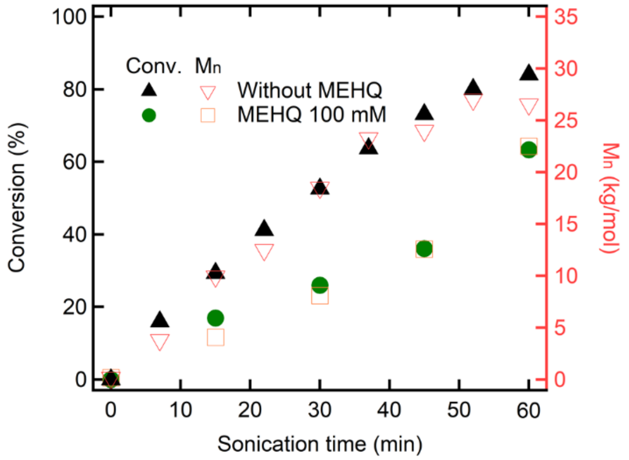

Figure S6. Conversions and molecular weight under continuous ultrasonic irradiation of HEA without MEHQ ([HEA] = 0.8 M, $[HEA]_0/[TTC]_0 = 300$) and with MEHQ ([HEA] = 0.8 M, $[HEA]_0/[TTC]_0 = 300$, $[MEHQ]_0 = 100$ mM). The polymerization rate decreased in the presence of MEHQ in aqueous microdroplets.

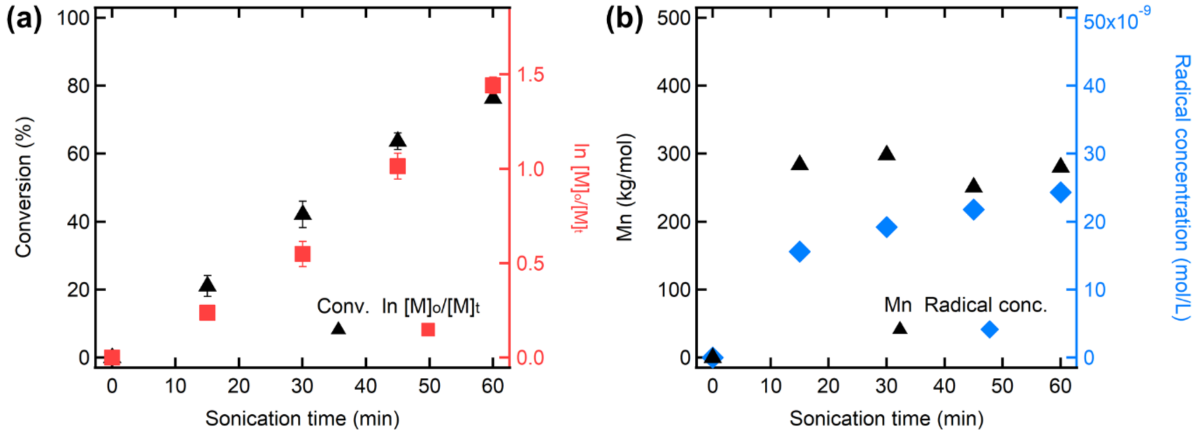

Figure S7. (a) Reaction kinetics of free radical polymerization of HEA ($[HEA]_0$ = 0.8 M). (b) Active growing radical concentration roughly calculated under the assumption that the initiation and termination rates are identical.

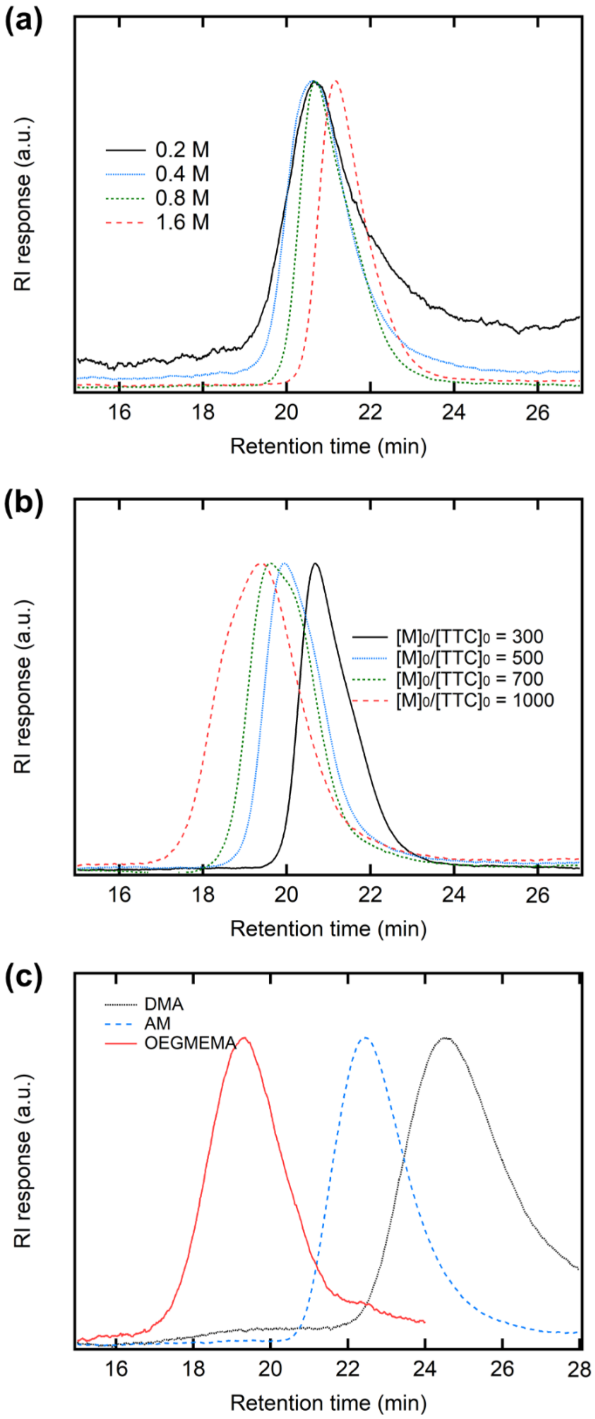

Figure S8. DMF SEC chromatographs of the synthesized polymers after 1 h sonication. The effects of reaction conditions of (a) $[HEA]_0$ ($[HEA]_0/[TTC]_0$ = 300), (b) $[HEA]_0/[TTC]_0$ ($[HEA]_0$ = 0.8M), and (c) types of monomers were presented. DMA = N,N-Dimethylacrylamide, AM = 4-Acryloylmorpholine, OEGMEMA = oligo(ethylene glycol) methyl ether methacrylate.

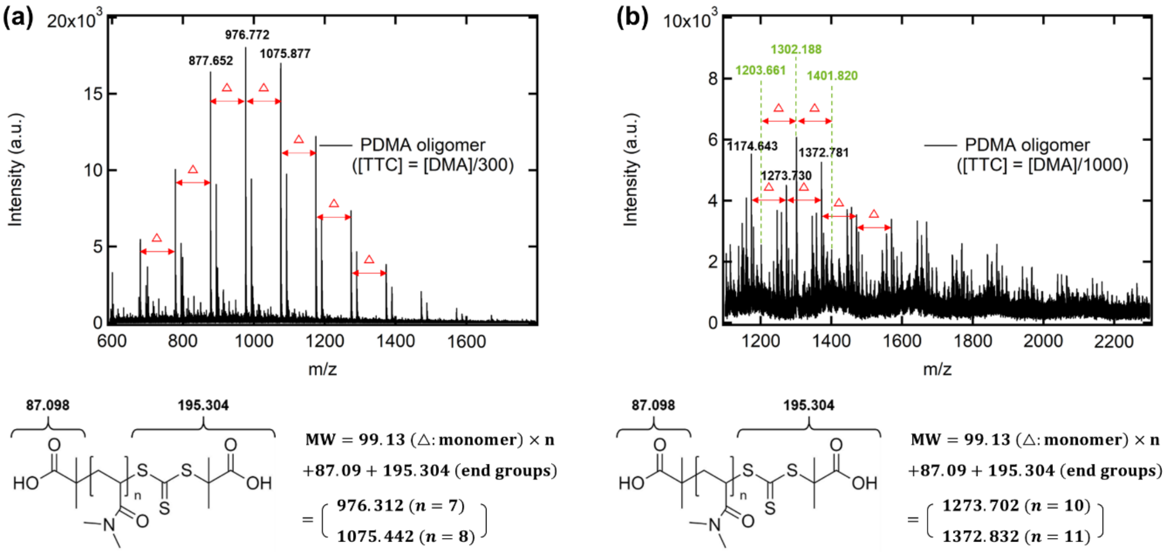

Entry	[M]	$[M]_0/[TTC]_0$	time	Conv.	$M_{n,th}$	$M_{n,SEC}$	$M_{n,MALDI}$
(a)	0.8 M	300	5 min	3.0 %	1,170 Da	-	1,010 Da
(b)	0.8 M	1000	5 min	1.9 %	2,490 Da	-	2,200 Da

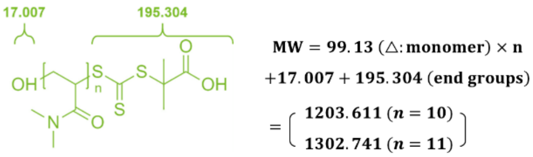

Figure S9. MALDI traces of PDMA oligomers over the range (a) 600-1800 Da and (b) 1100-2300 Da (Matrix: t-3-indole acrylic acid, no salt). The oligomers were synthesized via microdroplet-mediated radical polymerization. To make oligomers, which is suitable for MALDI measurement, we turned off polymerization reaction after 5 min sonication. Calculated number average molecular weight by MALDI ($M_{n,MALDI} = \sum_{i=1}^{N} N_i M_i / \sum_{i=1}^{N} N_i$) was consistent with $M_{n,th}$ ($M_{n,th}$ = conversion × $[M]_0/[TTC]_0$ × $MW_{monomer}$ + MW_{TTC}).

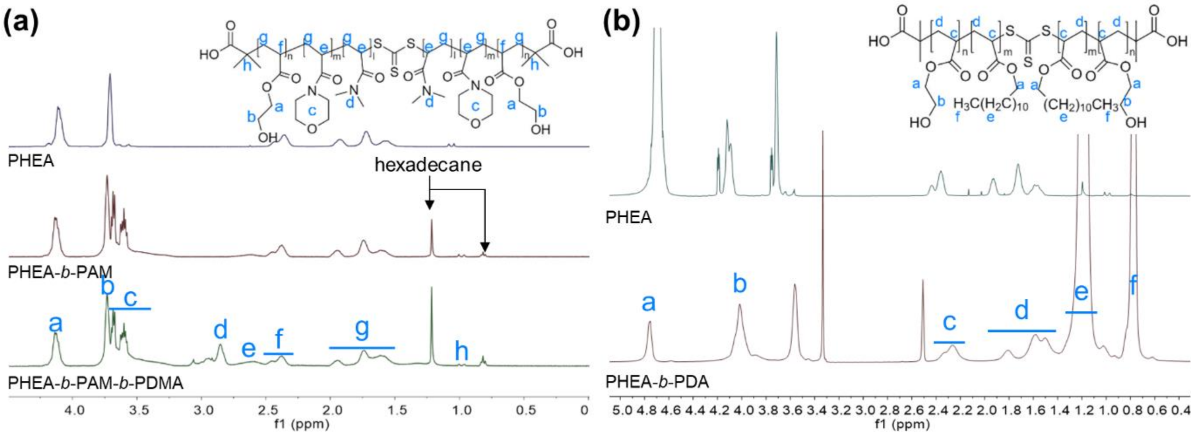

Figure S10. (a) ^{1}H NMR spectra of PHEA, PHEA-*b*-PAM, and PHEA-*b*-PAM-*b*-PDMA (400 MHz, D_2O, 25 ℃) (300 equivalent per TTC, $[HEA]_0$ = 0.8 M, 60 min sonication time at each stage). (b) ^{1}H NMR spectra of PHEA and PHEA-*b*-PDA (400 MHz, DMSO-d_6, 25 ℃).

Supporting References

(1) Coca S.; Jasieczek C. B.; Beers K. L.; Matyjasewski K. Polymerization of Acrylates by Atom Transfer Radical Polymerization. Homopolymerization of 2-Hydroxyethyl Acrylate, J. Polym. Sci. A Polym. Chem. **1998**, 36, 1417-1424.

(2) Lai J. T.; Filla D.; Shea R. Functional Polymers from Novel Carboxyl-Terminated Trithiocarbonates as Highly Efficient RAFT Agents, Macromolecules **2002**, 35, 6754-6756.

(3) Hochanadel C. J. Effect of cobalt γ-radiation on water and aqueous solutions, J. Phys. Chem. **1952**, 56, 587-594.

(4) Awtrey A. D.; Connick R. E. The Absorption Spectra of I_2, I_3^-, I^-, IO_3^-, $S_4O_6^=$ and $S_2O_3^=$. Heat of the Reaction $I_3^- = I_2 + I^-$, J. Am. Chem. Soc. **1951**, 73, 1842-1843.

(5) Berry J. D.; Neeson M. J.; Dagastine R. R.; Chan D. Y. C.; Tabor R. F. Measurement of surface and interfacial tension using pendant drop tensiometry, J. Colloid Interface Sci. **2015**, 454, 226-237.
