# Supplementary material for: Microdroplet-Mediated Radical Polymerization
Source: ACS Cent Sci. 2022 Aug 12;8(9):1265–71. doi: 10.1021/acscentsci.2c00694 (PMC9523774; doi:10.1021/acscentsci.2c00694)
Supplement: Supplementary file 2 — oc2c00694_si_002.pdf [file oc2c00694_si_002.pdf]

oc-2022-00694a.R1

Name: Peer Review Information for "Microdroplet-Mediated Radical Polymerization"

First Round of Reviewer Comments

Reviewer: 1

Comments to the Author

I applaud this accomplishment by the authors which demonstrates the power of microdroplet chemistry to effect interesting chemical transformations. This paper is well written and although I would have liked the authors to credit Lee et at. [LEE, J. K., KIM, S., NAM, H. G. & ZARE, R. N. (2015). Microdroplet fusion mass spectrometry for fast reaction kinetics. Proceedings of National Academy of Sciences, USA 112(13), 3898–3903] as the first quantitative demonstration of reaction rate acceleration in water microdroplets, this is an unnecessary detail but it would be a kindness to Lee who has just joined the faculty of SNU. In the area of polymer chemistry, I regard this work as a major advance.

Reviewer: 2

Comments to the Author

The authors utilize the special properties of microdroplet interfaces to successfully synthesize hydrophilic, hydrophobic, amphiphilic polymers as well as block copolymers. The experiment is related to chemical reaction acceleration at the air-liquid interface of microdroplets, in which scaled up synthesis can be performed by recycling droplets[1]. One bottleneck of using spray-generated microdroplets for synthesis is their short lifetime (millisecond scale), which is circumvented by recycling the liquid and reforming microdroplets or - as here - by creating aqueous microdroplets confined in oil phase to extend the reaction time (up to hours in this work). The authors provide evidence that hydroxyl radicals, spontaneously generated near the water-oil interface, initiate polymerization, and monomers are then involved in chain propagation. The synthesis of hydrophobic polymers validates the suggestion that the outer interface (in the oil phase) is involved and supports the proposal that hydroxyl radicals are transferred to the aqueous microdroplet interface. Since microdroplets are formed upon sonication, the polymerization progress can be initiated or paused by switching the system ‘on’ (sonication) or ‘off’ (centrifugal merging), providing an easy way to control copolymer synthesis.

Positive aspects:

1. This work describes a simple but apparently versatile route to polymer synthesis. By using the endogenous radicals to initiate polymerization, the reliance on specific initiators and their triggering is greatly reduced, suggesting the broad applicability of this method. Also, the ‘on/off’ control can be used to easily introduce structural diversity into the synthesized polymers, which helps build up functional macromolecules with complex structures.

2. This work harnesses the usual advantages of radical-based polymerization, maintenance of a population of radicals, to maximize synthesis. In related work [2–4], in which radicals are used for redox reactions in droplets, there are no radical chain reactions as there are here.

Concerns:

1. The characterization of aqueous droplets generated upon sonication could be more comprehensive. Based on the inset image of Figure 2a, the aqueous droplets display quite variable sizes, making the averaged droplet size too general to characterize the whole population. This is especially true given the literature evidence[5] that reaction rates (at least in spray-generated microdroplets) vary inversely with droplet size. The size distribution should be measured.

2. Based on Figure S1b, the spectroscopic method offers a dynamic range of H_2O_2 quantification up to 500 µM. However, the suggested concentrations of H_2O_2 generated in microdroplets without N_2 purging (Figure 2b) are much higher than that (millimolar scale). The calibration curve should extend over the concentration range of the measurements to be reliable.

3. Replicate measurements are required for Figure 2c to reach reliable conclusions on reaction order. Since Figure 2c and Figure S6a describe kinetics of the same system (HEA polymerization), their disagreement suggests poor measurement reproducibility. For example, at 60 min, Figure 2c indicates the $\ln[M]_0/[M]_t$ to be around 1.8 while the corresponding value at Figure S6a is around 1.5

4. More justification is required for the statement 'it did not follow the first-order kinetics for the monomer concentrations typically observed in controlled radical polymerizations' (page 3, right column, line 38). No information is given on how the dotted line was plotted in Figure 2c. The starting four data points indicate a linear relationship, does this contrast with the 'non-first order' statement?

5. Figure S2 is not found in SI. As described at the section 'Interfacial tension measurement' in the SI, the interfacial tension was measured by analyzing droplet shapes. Do droplets generated during the measurement have comparable sizes to those generated upon sonication? The statement 'the reduced tension at the oil/water interface in the presence of HEA' (page 3, right column, line 41) suggests different shapes of pure aqueous droplets and monomer-dissolved droplets during interfacial tension measurement. If so, why do the two droplets share close droplet sizes as indicated in Figure 2a?

6. Based on Figure 3a, reaction rates (the line slopes) during the three 'on' segments are different. Is this general? If so, what are possible reasons to slow down chain growth in the second 'on' segment?

7. The suggestion of a relationship to polymer synthesis in living systems is not based on any evidence.

8. The process whereby hydroxyl radicals are generated should be discussed further. The authors discuss an 'interfacial energy' without explanation.

Minor points:

9. Figure S9 is inserted among the references while there are three blank pages in the SI.

10. The authors attribute the disagreement between $M_{n,SEC}$ and $M_{n,th}$ of PAM and PDMA (entry #8 &9 in Table 1) to 'variation in hydrodynamic sizes of the polymers in the eluent' (page 4, left column, line 36). Can the authors provide a more detailed explanation of this, for example, how structural features impact their hydrodynamic sizes?

References:

(1) Nie, H.; Wei, Z.; Qiu, L.; Chen, X.; Holden, D. T.; Cooks, R. G. High-Yield Gram-Scale Organic Synthesis Using Accelerated Microdroplet/Thin Film Reactions with Solvent Recycling. Chem. Sci. 2020, 11 (9), 2356–2361. https://doi.org/10.1039/c9sc06265c.

(2) Lee, J. K.; Walker, K. L.; Han, H. S.; Kang, J.; Prinz, F. B.; Waymouth, R. M.; Nam, H. G.; Zare, R. N. Spontaneous Generation of Hydrogen Peroxide from Aqueous Microdroplets. Proc. Natl. Acad. Sci. U. S. A. 2019, 116 (39), 19294–19298. https://doi.org/10.1073/pnas.1911883116.

(3) Lee, J. K.; Samanta, D.; Nam, H. G.; Zare, R. N. Micrometer-Sized Water Droplets Induce Spontaneous Reduction. J. Am. Chem. Soc. 2019, 141 (27), 10585–10589. https://doi.org/10.1021/jacs.9b03227.

(4) Qiu, L.; Psimos, M. D.; Cooks, R. G. Spontaneous Oxidation of Aromatic Sulfones to Sulfonic Acids in Microdroplets. J. Am. Soc. Mass Spectrom. 2022. https://doi.org/10.1021/jasms.2c00029.

(5) Wei, Z.; Li, Y.; Cooks, R. G.; Yan, X. Accelerated Reaction Kinetics in Microdroplets: Overview and Recent Developments. Annu. Rev. Phys. Chem. 2020, 71, 31–51. https://doi.org/10.1146/annurev-physchem-121319-110654.

Author's Response to Peer Review Comments:

ACS Central Science – oc-2022-00694a

Submission date: Jun 13, 2022

Dear Dr. Editor and reviewers:

Thank you for inviting us to submit a revised draft of our manuscript entitled, “Microdroplet-Mediated Radical Polymerization” to ACS Central Science. We also appreciate the time and effort you and each of the reviewers have dedicated to providing insightful feedback on ways to strengthen our paper. Thus, it is with great pleasure that we submit our revised manuscript for further consideration. We have incorporated changes that reflect the detailed suggestions you have graciously provided. We also hope that our edits and the responses we provide below satisfactorily address all the issues and concerns you and the reviewers have noted.

To facilitate your review of our revisions, the following is a point-by-point response to the questions and comments delivered in your letter dated July 06, 2022.

Again, thank you for giving us the opportunity to strengthen our manuscript with your valuable comments and queries. We have worked hard to incorporate your feedback and hope that these revisions persuade you to accept our submission.

Sincerely,

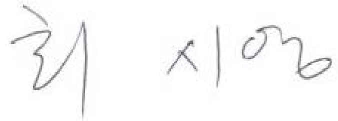

Siyoung Q. Choi
Associate Professor
Department of Chemical and Biomolecular Engineering
Korea Advanced Institute of Science and Technology

sqchoi@kaist.ac.kr

(Tel)+82-42-350-3914

Reviewer: 1

Recommendation: Reconsider after major revisions noted.

(1) Comment	I applaud this accomplishment by the authors which demonstrates the power of microdroplet chemistry to effect interesting chemical transformations. This paper is well written and although I would have liked the authors to credit Lee et at. [LEE, J. K., KIM, S., NAM, H. G. & ZARE, R. N. (2015). Microdroplet fusion mass spectrometry for fast reaction kinetics. Proceedings of National Academy of Sciences, USA 112(13), 3898–3903] as the first quantitative demonstration of reaction rate acceleration in water microdroplets, this is an unnecessary detail but it would be a kindness to Lee who has just joined the faculty of SNU. In the area of polymer chemistry, I regard this work as a major advance.
(1) Response	Thank you for your comments and suggestions. We changed reference and added the sentence that Lee et al. (LEE, J. K., KIM, S., NAM, H. G. & ZARE, R. N. (2015). Microdroplet fusion mass spectrometry for fast reaction kinetics. Proceedings of National Academy of Sciences, USA 112(13), 3898–3903) was first quantitative demonstration of reaction rate acceleration in water microdroplets in the first paragraph of INTRODUCTION.
Revised sentence	Compared to bulk-phase-mediated reactions, microdroplet-mediated reactions can enhance the reaction rate drastically by factors of $\geq 10^3$.[1-5] The first quantitative demonstration of accelerated chemical reaction rate in water microdroplets was reported by Lee and co-workers.[5]

Reviewer: 2

Recommendation: Publish in ACS Central Science after minor revisions noted.

Comments:

The authors utilize the special properties of microdroplet interfaces to successfully synthesize hydrophilic, hydrophobic, amphiphilic polymers as well as block copolymers. The experiment is related to chemical reaction acceleration at the air-liquid interface of microdroplets, in which scaled up synthesis can be performed by recycling droplets[1]. One bottleneck of using spray-generated microdroplets for synthesis is their short lifetime (millisecond scale), which is circumvented by recycling the liquid and reforming microdroplets or - as here - by creating aqueous microdroplets confined in oil phase to extend the reaction time (up to hours in this work). The authors provide evidence that hydroxyl radicals, spontaneously generated near the water-oil interface, initiate polymerization, and monomers are then involved in chain propagation. The synthesis of hydrophobic polymers validates the suggestion that the outer interface (in the oil phase) is involved and supports the proposal that hydroxyl radicals are transferred to the aqueous microdroplet interface. Since microdroplets are formed upon sonication, the polymerization progress can be initiated or paused by switching the system 'on' (sonication) or 'off' (centrifugal merging), providing an easy way to control copolymer synthesis.

Positive aspects:

1. This work describes a simple but apparently versatile route to polymer synthesis. By using the endogenous radicals to initiate polymerization, the reliance on specific initiators and their triggering is greatly reduced, suggesting the broad applicability of this method. Also, the 'on/off' control can be used to easily introduce structural diversity into the synthesized polymers, which helps build up functional macromolecules with complex structures.

2. This work harnesses the usual advantages of radical-based polymerization, maintenance of a population of radicals, to maximize synthesis. In related work [2–4], in which radicals are used for redox reactions in droplets, there are no radical chain reactions as there are here.

References:
(1) Nie, H.; Wei, Z.; Qiu, L.; Chen, X.; Holden, D. T.; Cooks, R. G. High-Yield Gram-Scale Organic Synthesis Using Accelerated Microdroplet/Thin Film Reactions with Solvent Recycling. Chem. Sci. 2020, 11 (9), 2356–2361. https://doi.org/10.1039/c9sc06265c.
(2) Lee, J. K.; Walker, K. L.; Han, H. S.; Kang, J.; Prinz, F. B.; Waymouth, R. M.; Nam, H. G.; Zare, R. N. Spontaneous Generation of Hydrogen Peroxide from Aqueous Microdroplets. Proc. Natl. Acad. Sci. U. S. A. 2019, 116 (39), 19294–19298. https://doi.org/10.1073/pnas.1911883116.
(3) Lee, J. K.; Samanta, D.; Nam, H. G.; Zare, R. N. Micrometer-Sized Water Droplets Induce Spontaneous Reduction. J. Am. Chem. Soc. 2019, 141 (27), 10585–10589. https://doi.org/10.1021/jacs.9b03227.
(4) Qiu, L.; Psimos, M. D.; Cooks, R. G. Spontaneous Oxidation of Aromatic Sulfones to Sulfonic Acids in Microdroplets. J. Am. Soc. Mass Spectrom. 2022. https://doi.org/10.1021/jasms.2c00029.

(1) Comment	The characterization of aqueous droplets generated upon sonication could be more comprehensive. Based on the inset image of Figure 2a, the aqueous droplets display quite variable sizes, making the averaged droplet size too general to characterize the whole population. This is especially true given the literature evidence[5] that reaction rates (at least in spray-generated microdroplets) vary inversely with droplet size. The size distribution should be measured.<br><br>Reference:<br>(5) Wei, Z.; Li, Y.; Cooks, R. G.; Yan, X. Accelerated Reaction Kinetics in Microdroplets: Overview and Recent Developments. Annu. Rev. Phys. Chem. 2020, 71, 31–51. https://doi.org/10.1146/annurev-physchem-121319-110654.
(1) Response	Thank you for point it out. We analyzed size distributions of aqueous microdroplets and added it to Revised Figure S1.
Revised Figure	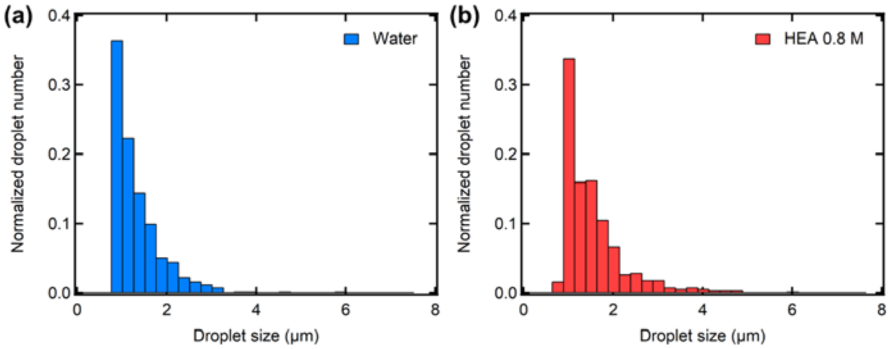<br><br>Figure S1. Size distribution of the microdroplets generated by ultrasonic emulsification of 10:1 (v/v) mixtures of hexadecane oil and (a) pure water or (b) HEA solution at 30 min sonication time.

<table>
<tr><td>(2) Comment</td><td>Based on Figure S1b, the spectroscopic method offers a dynamic range of H_2O_2 quantification up to 500 μM. However, the suggested concentrations of H_2O_2 generated in microdroplets without N_2 purging (Figure 2b) are much higher than that (millimolar scale). The calibration curve should extend over the concentration range of the measurements to be reliable.</td></tr>
<tr><td>(2) Response</td><td>We measured concentration of H_2O_2 generated in microdroplets without N_2 purging after dilution of collected water solutions. For example, concentration of H_2O_2 (without N_2 purging) was about 10 mM at 20 min sonication in Figure 2b, and this value was analyzed by following steps:
1. Dilute water phase by factors of 1/100 after desired sonication time.
2. Measure H_2O_2 concentration by UV-Vis spectroscopy.
3. Calculated concentration of H_2O_2 was about 100 μM.
4. Thus, the concentration of H_2O_2 before dilution is about 10 mM.
We added this comment in the caption of Figure S1 (Revised: Figure S2).</td></tr>
<tr><td>Revised Figure caption</td><td>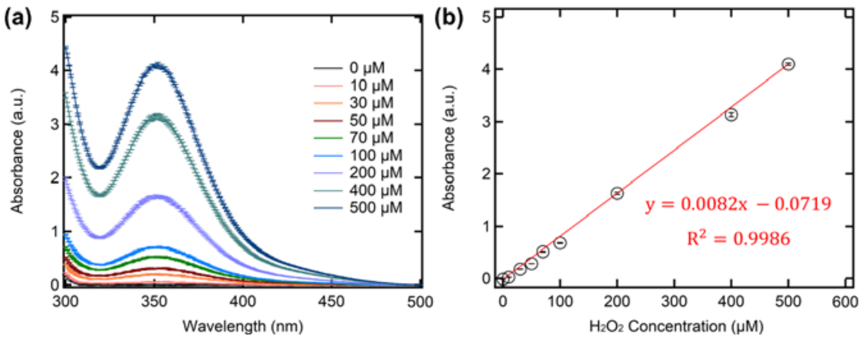

Figure S2. Intensity of the UV-visible absorption according to the H_2O_2 concentration in a bulk water. The I^- ion is oxidized to I^{3-} ion from the catalytic activity of ammonium molybdite under the presence of H_2O_2, and absorption peak occurs at 353 nm. (a) UV-vis spectra of the solutions with varying concentration of H_2O_2. (b) Linear increase of absorbance peak at 353 nm with increase of H_2O_2 concentration. Higher concentrations above 500 μM were measured after dilution.</td></tr>
</table>

<table>
<tr><td>(3) Comment</td><td>Replicate measurements are required for Figure 2c to reach reliable conclusions on reaction order. Since Figure 2c and Figure S6a describe kinetics of the same system (HEA polymerization), their disagreement suggests poor measurement reproducibility. For example, at 60 min, Figure 2c indicates the $\ln[M]_0/[M]_t$ to be around 1.8 while the corresponding value at Figure S6a is around 1.5</td></tr>
<tr><td>(3) Response</td><td>We checked reproducibility several times, and results were similar. We added error bar in Figure 2c and Figure S6a (Revised: S7a). The discrepancy between Figure 2c and Figure S6a (Revised: S7a) might be due to the different polymerization systems. Figure 2c shows “RAFT polymerization”, while Figure S6a (Revised: S7a) shows “free radical polymerization (FRP)”.
Although kinetics of FRP and RAFT are similar in general polymerization, generated molecular weights (MW) are different. MW linearly increases with conversion in RAFT polymerization, while MW is independent with conversion in free radical polymerization. At 1 h sonication, MW of the synthesized polymers was ~30,000 g/mol in Figure 2c and was ~250,000 g/mol in Figure S6a. We also added MW data in Figure S6b (Revised: S7b).
High MW polymer solutions have high viscosity (Mark-Houwink equation: [η] = KMa, where [η]: intrinsic viscosity, M: molecular weight, K,a: Mark-Houwink parameters), thus polymer solutions synthesized by FRP would have higher viscosity than polymer solutions synthesized by RAFT. In our systems, the difference in viscosity was evident to the naked eye. Since the definition of viscosity is resistance to deformation, the high solution viscosity in FRP could prevent formation of emulsion droplets, which initiate polymerization. This may explain why the kinetics in Figure S6a (Revised: S7a) (FRP) are slightly slower than in Figure 2c (RAFT) although the kinetics of FRP and RAFT are similar in general polymerizations.</td></tr>
<tr><td>Revised Figure</td><td>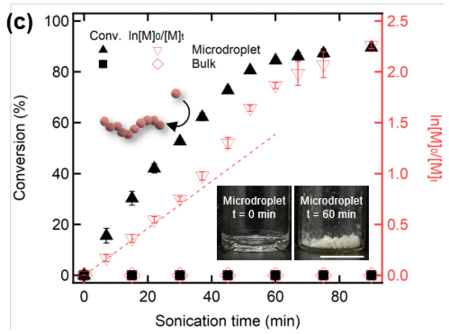

Figure 2. (c) Polymerization of HEA within the aqueous microdroplets closed by hexadecane oil ([HEA] = 0.8 M, [HEA]:[TTC] = 300:1). Conversion linearly increase under continuous ultrasonic irradiation, not following first-order kinetics (dotted line, generated by the first two points). The bulk HEA solution data is also presented as a reference. The inset images are the remaining substance after removal of solvents (scale bar: 1 mm).</td></tr>
</table>

<table>
<tr>
<td>Revised Figure</td>
<td>
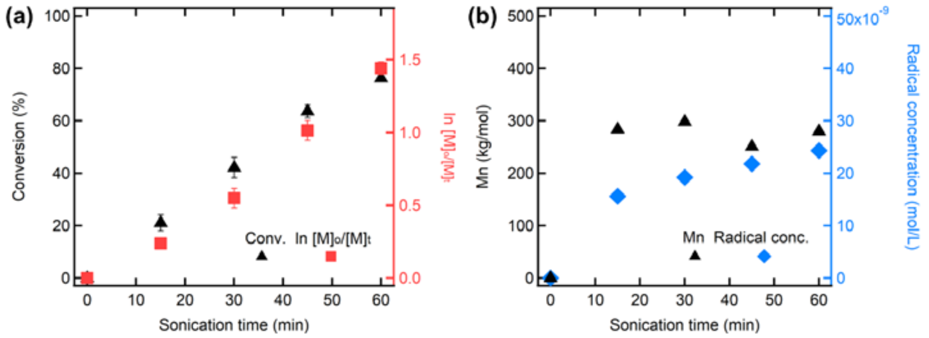

Figure S7. (a) Reaction kinetics of free radical polymerization of HEA ($[HEA]_0$ = 0.8 M). (b) Active growing radical concentration roughly calculated under the assumption that the initiation and termination rates are identical.
</td>
</tr>
</table>

(4) Comment	More justification is required for the statement 'it did not follow the first-order kinetics for the monomer concentrations typically observed in controlled radical polymerizations' (page 3, right column, line 38). No information is given on how the dotted line was plotted in Figure 2c. The starting four data points indicate a linear relationship, does this contrast with the 'non-first order' statement?
(4) Response	● We added the statement that how the dotted line was plotted in the caption of Figure 2c. We generated dotted line by the first two points.<br>● We did not understand the exact origin of non-first order kinetics. However, the first four data points may appear to follow a linear relationship because of the following hypothesis.<br>- Under the steady-state assumption, $R_p = -d[M]/dt = k_p[M][R\cdot]_{st-st}$, where k_p is the propagation rate constant, and $[R\cdot]_{st-st}$ is the concentration of the steady state active radical chains. Rearrange the above equation: $\ln\frac{[M]_0}{[M]_t} = k_p[R\cdot]_{st-st}t$ or $[M]_t = [M]_0 e^{-k_p[R\cdot]_{st-st}t}$.<br>- Thus, polymerization kinetic shows first-order only when $[M]_t$ decreases exponentially. Small deviation in $[M]_t$ has little effect on $\ln\frac{[M]_0}{[M]_t}$ near the starting points of polymerization. However, this small deviation can have a substantial effect on $\ln\frac{[M]_0}{[M]_t}$ as the reaction proceeds. Because of this, the first four data points may appear to follow a linear relationship.
Revised Figure caption	Figure 2. (c) Polymerization of HEA within the aqueous microdroplets closed by hexadecane oil ([HEA] = 0.8 M, [HEA]:[TTC] = 300:1). Conversion linearly increase under continuous ultrasonic irradiation, not following first-order kinetics (dotted line, generated by the first two points). The bulk HEA solution data is also presented as a reference. The inset images are the remaining substance after removal of solvents (scale bar: 1 mm).

(5) Comment	Figure S2 is not found in SI. As described at the section 'Interfacial tension measurement' in the SI, the interfacial tension was measured by analyzing droplet shapes. Do droplets generated during the measurement have comparable sizes to those generated upon sonication? The statement 'the reduced tension at the oil/water interface in the presence of HEA' (page 3, right column, line 41) suggests different shapes of pure aqueous droplets and monomer-dissolved droplets during interfacial tension measurement. If so, why do the two droplets share close droplet sizes as indicated in Figure 2a?
(5) Response	● Thank you for pointing it out. We revised and rearranged figures in SI.<br>● Droplet size during pendant drop measurement was ~ 1 mm in diameter, which is quite different with microdroplet diameter ~ 1 μm. However, surface tension is normally independent of size unless the size becomes molecular scale. Since ~ 1 μm droplet is not molecular scale, surface tension between ~ 1 mm droplet and ~ 1 μm would be similar. Experimentally, measuring the surface tension of a droplet with a diameter of ~ 1 μm was quite challenging.<br>● There might be two possible reasons for close droplet sizes between "pure aqueous droplet" and "aqueous droplet containing HEA".<br>1. HEA is not surfactant. Though it can reduce surface tension in Figure S2 (Revised: Figure S3), the difference is insignificant. Thus, it may not change droplet size significantly.<br>2. It might be a result of the unstable conditions. When we make aqueous microdroplet with 10 mM Span80 in hexadecane oil (Surface tension < 5mN/m), its droplet diameter was >> 1 μm after 24h of droplet generation (see below OM image). This implies that the stable droplet diameter would be much larger than 1 μm for 0.8 M HEA solution (surface tension ~ 30 mM/m, in Figure S2 (Revised: Figure S3)). Thus, droplet sizes that we measured right after sonication were under the unstable state which was dominated by ultrasound energy, not by surface tension.<br>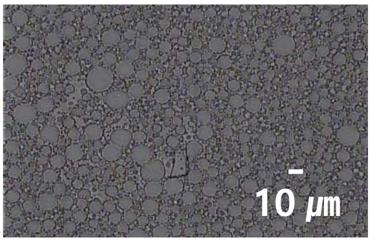<br><br>Aqueous droplets in hexadecane (Span80 10 mM) after 24h of droplet generation by ultrasound.

<table>
<tr><td>(6) Comment</td><td>Based on Figure 3a, reaction rates (the line slopes) during the three ‘on’ segments are different. Is this general? If so, what are possible reasons to slow down chain growth in the second ‘on’ segment?</td></tr>
<tr><td>(6) Response</td><td>We performed multiple on/off experiments and added error bar in Figure 3a. Although we do not know exact reason, these phenomena generally reported during on/off procedures (ref 1-3). This may be a result of the short interval between ‘on’ and ‘off’ procedures. Due to the short interval, variance of conversions appears to be big, but this effect might be negligible for long-term reactions.

References:
(1) McKenzie T. G.; Colombo E.; Fu Q.; Ashokkumar M.; Qiao G. G. Sono-RAFT polymerization in Aqueous Medium, Angew. Chem. Int. Ed., 2017, 56, 12302-12306. https://doi.org/10.1002/anie.201706771.
(2) Zhang Z.; Zeng T.-Y.; Xia L.; Hong C.-Y.; Wu D.-C.; You Y.-Z. Synthesis of polymers with on-demand sequence structures via dually switchable and interconvertible polymerizations, Nat. Commun., 2018, 9, 2577-2585. https://doi.org/10.1038/s41467-018-05000-2.
(3) Luo J.; Li M.; Xin M.; Sun W.; Xiao W. Visible Light Induced RAFT Polymerization of 2-Vinylpyridine without Exogenous Initiators or Photocatalysts, Macromol. Chem. Phys. 2016, 217, 1777−1784. https://doi.org/10.1002/macp.201600141.</td></tr>
<tr><td>Revised Figure</td><td>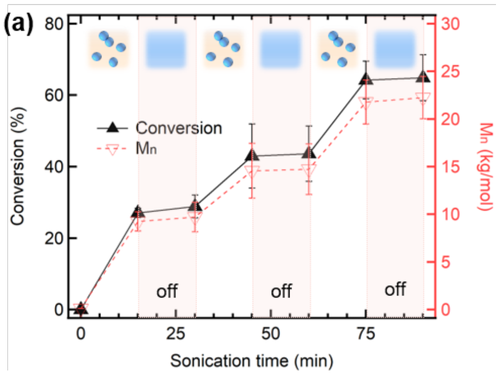

Figure 3. (a) Monomer conversion and molecular weight with altering ultrasonication (“on”) and centrifugal merging (“off”). The 1:10 (v/v) mixture of the HEA aqueous solution (300 equivalent per TTC, [HEA] = 0.8 M) and hexadecane oil was used.</td></tr>
</table>

(7) Comment	The suggestion of a relationship to polymer synthesis in living systems is not based on any evidence.
(7) Response	We deleted the suggestions of a relationship to polymer synthesis in living systems.

(8) Comment	The process whereby hydroxyl radicals are generated should be discussed further. The authors discuss an ‘interfacial energy’ without explanation.
(8) Response	● We added further discussion about interfacial energy in the first paragraph of “Comparative study of microdroplet-mediated radical polymerization” section.<br>● We also suggested possible reason in the first paragraph of RESULT AND DISCUSSION (in the first draft). “It was motivated by Xiong and co-workers, who reported the observation of a strong electric field ($\vec{E} \approx 10^7$ V/cm) at the oil-confined microdroplet surface. This electric field strength is sufficient to produce hydroxyl radicals from hydroxide ions, which readily recombine to form H_2O_2”.
Revised sentence	However, in our microdroplet-mediated polymerization, interfacial energy between two immiscible liquids was exploited to generate radicals. A strong electric field ($\vec{E} \approx 10^7$ V/cm) at the oil-confined microdroplet surface[11] produced hydroxyl radicals from hydroxide ions[9,10] under mild conditions.

(9) Comment	Figure S9 is inserted among the references while there are three blank pages in the SI.
(9) Response	Thank you for pointing it out. We removed blank pages and re-arranged SI figures.

(10) Comment	The authors attribute the disagreement between $M_{n,SEC}$ and $M_{n,th}$ of PAM and PDMA (entry #8 & 9 in Table 1) to 'variation in hydrodynamic sizes of the polymers in the eluent' (page 4, left column, line 36). Can the authors provide a more detailed explanation of this, for example, how structural features impact their hydrodynamic sizes?
(10) Response	If polymer-polymer interaction is much larger than polymer-solution interaction (possibly due to some functional groups), the size of the polymer would be reduced, and vice versa. Thus, hydrodynamic size of polymers is strongly dependent on solvents. For instance, in theta solvent, $R \sim N^{1/2}c^{0}$, while $R \sim N^{1/2}c^{-0.12}$ in good solvent. (ref 1) (R: polymer radius, N: degree of polymerization, c: concentration)<br>The disagreement between $M_{n,SEC}$ and $M_{n,th}$ of PAM and PDMA is a result of different hydrodynamic size with PMMA standard because SEC measure MW by their hydrodynamic size. When we analyze MW of PDMA by MALDI measurement, which analyze MW by mass, it showed quite similar value with $M_{n,th}$ in Figure S8 (Revised: Figure S9).<br><br>References:<br>(1) Gennes, P.-G. D. (1979). Scaling concepts in polymer physics. Ithaca, N.Y., Cornell University Press.

oc-2022-00694a.R2

Name: Peer Review Information for "Microdroplet-Mediated Radical Polymerization"

Second Round of Reviewer Comments

Reviewer: 2

Comments to the Author

As noted in my original review, this study is distinctive amongst papers on reaction acceleration in microdroplets in dealing with a free radical propagation reaction. I am not convinced - now that there are error bars in Fig 3 - that the on/off behavior has been fully established but leave it to the authors to reconsider this data.

Author's Response to Peer Review Comments:

ACS Central Science – oc-2022-00694a.R1

Original Submission Date: Jun 13, 2022

Dear Dr. Editor and reviewers:

We are submitting our revised manuscript entitled “Microdroplet-Mediated Radical Polymerization”, by Kyoungmun Lee, Hyun-Ro Lee, Young Hun Kim, Jaemin Park, Suchan Cho, Sheng Li, Myungeun Seo, and Siyoung Q. Choi, for publication in ACS Central Science.

We really appreciate all the comments and critiques from you and two reviewers, and we believe that the comments made our work far clarifier.

The synopsis label was revised. In addition, our opinion on on/off behaviors was included in the response letter to address the concerns of Reviewer 2.

We now feel the current revision has been strengthened considerably in response to the reviewers’ critiques, and are grateful for their close reading and would be grateful for your consideration. To facilitate your review of our revisions, the following is a point-by-point response to the questions and comments delivered in your letter dated July 28, 2022. We hope that you will be satisfied with our response and the revised manuscript. We thank you and the reviewers in advance.

Sincerely,

최 시영

Siyoung Q. Choi
Associate Professor
Department of Chemical and Biomolecular Engineering
Korea Advanced Institute of Science and Technology

sqchoi@kaist.ac.kr

(Tel)+82-42-350-3914

Reviewer: 2

Recommendation: Publish in ACS Central Science after minor revisions noted.

Comments:

As noted in my original review, this study is distinctive amongst papers on reaction acceleration in microdroplets in dealing with a free radical propagation reaction.

(1) Comment	I am not convinced - now that there are error bars in Fig 3 - that the on/off behavior has been fully established but leave it to the authors to reconsider this data.
(1) Response	We stand by the conclusion that the on/off polymerization behavior is established by dispersing (on) and merging (off) of the microdroplets as multiple repeats of the experiment consistently showed the same behavior.<br>Small variations in polymerization rate during each 'on' segment may be attributable to the variable polymerization medium as polymerization progresses, including monomer concentration, polymer concentration, average molecular weight, interfacial tension, and solution viscosity. In contrast to general homogeneous phase radical polymerizations, these have a complex effect on the polymerization rate in microdroplet-mediated radical polymerization. In the subsequent study, specific research will be discussed in depth.<br>Alternatively, the small transition of polymerization rate might be the result of experiment-to-experiment variation. To pause the polymerization, we need to manually remove the samples from the ultrasonic bath, then transport and load them into a centrifuge. This process requires a few minutes to complete, and during the time period, the reaction environment may change as any minute turbulence can cause the microdroplets to randomly merge. However, the observed variation does not change our main conclusion, as the on/off polymerization is clearly observed within each experiment.
